# Risk Prediction and New Prophylaxis Strategies for Thromboembolism in Cancer

**DOI:** 10.3390/cancers12082070

**Published:** 2020-07-27

**Authors:** Alice Labianca, Tommaso Bosetti, Alice Indini, Giorgia Negrini, Roberto Francesco Labianca

**Affiliations:** 1 Medical Oncology Unit, Department of Oncology and Haematology, ASST Papa Giovanni XXIII, 24121 Bergamo, Italy; alice.labiancadr@gmail.com (A.L.); tbosetti@asst-pg23.it (T.B.); gnegrini@asst-pg23.it (G.N.); 2 Medical Oncology Unit, Fondazione IRCCS Ca’ Granda Ospedale Maggiore Policlinico, 20019 Milano, Italy; alice.indini@gmail.com

**Keywords:** thromboprophylaxis, venous thromboembolism, chemotherapy, low-molecular-weight heparin (LMWH), VKA, UFH, DOACs

## Abstract

In the general population, the incidence of thromboembolic events is 117 cases/100,000 inhabitants/year, while in cancer patient incidence, it is four-fold higher, especially in patients who receive chemotherapy and who are affected by pancreatic, lung or gastric cancer. At the basis of venous thromboembolism (VTE) there is the so-called Virchow triad, but tumor cells can activate coagulation pathway by various direct and indirect mechanisms, and chemotherapy can contribute to VTE onset. For these reasons, several studies were conducted in order to assess efficacy and safety of the use of anticoagulant therapy in cancer patients, both in prophylaxis setting and in therapy setting. With this review, we aim to record principal findings and current guidelines about thromboprophylaxis in cancer patients, with particular attention to subjects with additional risk factors such as patients receiving chemotherapy or undergoing surgery, hospitalized patients for acute medical intercurrent event and patients with central venous catheters. Nonetheless we added a brief insight about acute and maintenance therapy of manifested venous thromboembolism in cancer patients.

## 1. Introduction

The correlation between cancer and venous thromboembolism (VTE), which comprehends deep vein thrombosis (DVT) and pulmonary embolism (PE), is well known. In the general population incidence of thromboembolic events is 117 cases/100,000 inhabitants/year, while in cancer patient incidence is four-fold higher and in patients who receive chemotherapy is seven-fold higher [1]. In addition, thromboembolic risk is higher in patients who receive hormonotherapy, who have central venous catheter and who undergo surgery.

At the basis of VTE there is the so-called Virchow triad (see Table 1).

The first data about the incidence of venous thromboembolism in oncological patients on active anticancer treatment come from NSABP-14 and NSABP-20 trials; in these trials, estrogen and progesterone receptors positive-nodes negative breast cancer patients treated with tamoxifen and chemotherapy had a higher incidence of VTE compared with patients receiving tamoxifen alone or placebo (5 years incidence 4.3%, 0.9% and 0.2%, respectively) [2,3]. Association of chemotherapy and anti-VEGF antibodies worsens even more the risk of venous and arterial thrombosis. In addition, patients with malignant gastric, pancreatic, pulmonary cancers or glioblastomas have a higher risk of VTE (10–30%) [4].

For these reasons, VTE prophylaxis in oncological patients has been the subject of several studies in the last years.

Recent international guidelines provided updated recommendations for the management of VTE in cancer patients. Nonetheless, several reviews have been published in the last few years: Horsted et al. in 2012 conducted a systematic review about the incidence of VTE in cancer patients, in order to provide data about the risk of VTE in different cancer types, stratify patients and highlight which kind of patient should receive prophylaxis [5]. In 2014 Matzdorff and colleagues outlined pathophysiology of VTE in cancer patients and reported the most recent indications about VTE therapy and prophylaxis, as well as the use of new oral anticoagulants (but concluded that DOACs were not recommended according to 2014 international guidelines) [6]. Singh et al. in 2017 outlined pathophysiology and diagnosis of venous thromboembolism in patients with cancer, as well as pharmacological and mechanical prophylaxis [7]; Imberti and colleagues, the subsequent year, reported the most important findings about VTE treatment, focusing on the results of Hokusai VTE-cancer trial about the comparison between low-molecular-weight heparin (LMWH) and the direct oral anticoagulant edoxaban, which showed that edoxaban is non-inferior to dalteparin with a trend toward fewer recurrent venous thromboembolic events, but with higher major bleeding risk [8].

Still, some uncertainties remain, both for prophylaxis and treatment of VTE, due to the yet limited evidence available. With this review, we aim to summarize the latest evidence on VTE prophylaxis and treatment in patients with cancer, based on the newest guidelines and papers published in the last few months, as well as synthesize the major clinical trials and meta-analyses that have been conducted until now and highlight the most clinically relevant unmet needs.

## 2. Thrombosis Pathophysiology in Cancer Patients

Tumor cells can activate coagulation pathway by a direct and an indirect mechanism: the direct mechanism involves the production of pro-coagulant factors such as the *tissue factor* which is constitutively expressed by tumor cells and which binds factor VII and activates coagulation pathway; and the *cancer procoagulant*, a cysteine protease expressed in tumor cells and in fetal tissues which activates factor X in absence of factor VII [9,10].

Among indirect mechanisms, we can enumerate production of cytokines such as IL-2, TNF and VEGF that activate monocytes, platelets and endothelial cells inducing procoagulant phenotype expression. In addition, tumor cells have superficial adhesion molecules that can bind monocytes, platelets and endothelial cells activating and stimulating fibrin production. Moreover, some predisposing factors can add prothrombotic risk such as hospitalization, systemic inflammatory status and tumor compression stasis.

Chemotherapy can contribute to VTE onset with various mechanisms, such as vessel walls acute damage, and this is the case of bleomycin, carmustine and vinca alkaloids or retarded damage for adriamycin; coagulation regulator factors reduction, such as reduction of protein C and S in the case of CMF scheme (cyclophosphamide, methotrexate and 5-fluorouracil) or reduction of antithrombin II by L-asparaginase.

## 3. VTE Risk Prediction in Cancer Patients

In order to assess VTE risk in cancer patients, various factors need to be considered. First of all, personal thrombophilic conditions such as advanced age, obesity, history of previous venous thromboembolism, prolonged immobility, prothrombotic blood alterations. Then, risk factors associated with the tumor itself, such as histology, grading, primary site, presence of metastasis, and factors associated with tumor treatment, such as ongoing or recent chemotherapy, hormonal therapy, anti-angiogenesis agents, immunotherapy, radiotherapy, recent surgery, presence of central venous catheters, hospitalization.

Some blood biomarkers have been studied and associated with elevated VTE risk, such as platelets and leucocytes count, D-dimer, soluble *P*-selectin and other markers of coagulation activation, markers of neutrophil extracellular trap formation, such as citrullinated histone H3 and many others [11,12,13].

Some risk models have been developed and proposed over the last few years. The most known and used is the Khorana risk score (Table 2) [14], published in 2008, validated in several subsequent studies and still used by clinicians. Some variations of the Khorana risk score have been published subsequently, such as the PROTECHT, CONKO and Vienna CATS score [15,16,17]. Thereafter, the COMPASS-CAT and the ONKOTEV56 models were developed, the first one for patients with breast, colorectal, lung and ovarian cancers, including variables such as cardiovascular risk factors, personal history of VTE, presence of central venous catheter, chemotherapy or hormonal therapy, tumor stage and platelet count; the second one based on Khorana risk score >2 associated with the presence of metastasis, vascular compression, previous VTE [18,19].

## 4. VTE Prophylaxis in Patients Receiving Chemotherapy

Clinical studies about the use of thromboprophylaxis in outpatients receiving systemic treatment for cancer were conducted in two moments; the first moment saw clinical trials evaluating safety and efficacy of low-molecular-weight heparin (LMWH) in this setting of patients, the second one evaluated safety and efficacy of direct oral anticoagulants (DOACs) in high risk patients.

Analyzing literature, five meta-analyses and two recent randomized trials investigated thromboprophylaxis in cancer patients. The five meta-analyses primarily analyzed LMWH use: in a meta-analysis published in 2012, Di Nisio et al. outlined how LMWH significantly reduces asymptomatic VTE (HR 0.54, CI 95% 0.38–0.75) with a non-statistically significant higher risk of major bleeding (HR 1.44, CI 95% 0.98–2.11) [20]. The 2014 meta-analysis of Ben-Aharon et al. showed similar results [21].

Two meta-analyses (Thein et al. and Fuentes et al.) [22,23] analyzed lung cancer setting; LMWH reduced the risk of VTE of about half, but it did not significantly affect OS. In Thein et al. meta-analysis, the use of LMWH significantly increased the risk of non-major, but clinically relevant bleeding (RR 3.35, CI 95% 2.09–5.06), while it did not affect the risk of major bleeding. In Fuentes et al. meta-analysis the use of LMWH did not significantly affect the risk of total bleedings.

The meta-analysis by Tun et al. analyzed only patients with advanced pancreatic cancer, demonstrating how the use of LMWH reduces the risk of symptomatic VTE (RR 0.18, 95% CI 0.08–0.39) without significantly increasing the rate of major bleeding (RR 1.25, 95% CI 0.48–3.31) [24].

Two randomized controlled trials were conducted in the last years to analyze the use of DOACs in patients receiving active treatment for cancer and with Khorana score ≥2 (see Table 2) [25,26].

The AVERT study, a double-blind, randomized, placebo-controlled clinical trial, evaluated the efficacy and safety of apixaban at a dosage of 2.5 mg twice/daily in the primary prevention of VTE in 574 ambulatory patients with Khorana score ≥2 receiving chemotherapy. Apixaban significantly reduced limbs deep vein thrombosis, pulmonary embolism and VTE-related death compared to placebo (4.2% vs. 10.2% with apixaban vs. placebo, respectively, HR 0.41, 95% CI 0.26–0.65). However, apixaban prophylaxis was associated with an increased risk of major bleedings (3.5% vs. 1.8% with apixaban vs. placebo, HR 2.00 CI 95% 1.01–3.95) [25].

The second clinical trial that assessed the efficacy and safety of a DOAC in the primary prevention of VTE was the CASSINI study, a randomized, double-blind clinical trial with rivaroxaban 10 mg/day vs. placebo, in 841 outpatients affected by stage III or IV tumors and Khorana score ≥2 which were about to start systemic chemotherapy. In this study, patients were screened with venous ultrasound before randomization and every eight weeks afterwards. The use of rivaroxaban was associated with a statistically insignificant decrease in the primary outcome in ITT (intention-to-treat) population (deep vein thrombosis of the limbs 6.0% vs. 8.8% with rivaroxaban vs. placebo, HR 0.66, CI 95% 0.4–1.9), while in a prespecified analysis of all randomized patients the primary VTE endpoint on treatment occurred in 2.6% of 420 and in 6.4% of 421 patients in the rivaroxaban and placebo arms, respectively (HR0.40, 95% CI 0.2–0.8); also, the use of rivaroxaban was associated with a statistically insignificant increase in the risk of major bleeding (2% vs. 1%, rivaroxaban vs. placebo arm, HR 1.96, CI 95% 0.59–6.49) [26].

In a recent meta-analysis published by Becattini et al. including Avert study, Cassini study and a phase II study with apixaban, thromboprophylaxis reduced the incidence of VTE of about 50% (49%, CI 95% 0.43–0.61) in particular in patients with lung cancer, pancreatic cancer and in the so-called high-risk patients, without observing a significant increase in the risk of major bleeding (OR 1.3, 95% CI 0.98–1.73) [27].

Some ad hoc studies evaluated the impact of VTE prophylaxis in patients affected by multiple myeloma in therapy with thalidomide or lenalidomide, and it was found that prophylactic dosage of LMWH, warfarin or aspirin reduced the risk of VTE; moreover, there was a lower effect of warfarin compared to LMWH in patients over 65 years [28,29].

In late 2019, an update of clinical practice guidelines from the International Initiative on Thrombosis and Cancer working group has been published. According to these guidelines, thromboprophylaxis with rivaroxaban or apixaban is recommended in ambulatory intermediate/high risk patients receiving chemotherapy and who do not have active bleeding or high bleeding risk. In particular, VTE prophylaxis is recommended in patients treated with immunomodulatory drugs, chemotherapy and steroids, due to the high risk of VTE in this setting of patients [30].

Thromboprophylaxis with LMWH is suggested in ambulatory patients with metastatic or locally advanced pancreatic cancer receiving chemotherapy and without major bleeding risk [31,32].

The American Society of Clinical Oncology (ASCO) recently updated its clinical practice guidelines. Among this update, the introduction of DOACs in the treatment strategy and in the prophylaxis of VTE in cancer patients represents one of the most important novelty [33,34].

A systematic review and meta-analysis which includes 30 randomized controlled trials and determines the efficacy and safety of thromboprophylaxis in patients with cancer has recently been published [35]. In this meta-analysis no significant difference in all-cause mortality has been observed in patients who did and did not receive thromboprophylaxis. Thromboprophylaxis can reduce VTE events in patients with cancer undergoing surgery or chemotherapy and does not increase major bleeding events or the incidence of thrombocytopenia. Limitations of this review are different cancer types and staging, different anticoagulants and dosage administered and potential interactions between antithrombotic drugs and patients’ concomitant medications.

A careful analysis of international guidelines allows us to summarize the following recommendations [34,36]:

- Anti-thrombotic prophylaxis should not be offered routinely in all unselected cancer patients on active oncological therapy;
- In high-risk patients with multiple myeloma and in therapy with lenalidomide or thalidomide, prophylaxis with LMWH should always be practiced unless specific clinical contraindications. In patients in this setting, but at low risk of VTE, aspirin prophylaxis can be practiced instead of LMWH;
- In general, prophylaxis with LMWH, apixaban or rivaroxaban should be considered for cancer outpatients who receive chemotherapy and who are at high thromboembolic risk.

Prospective randomized ad hoc trials evaluating thromboprophylaxis in different types of neoplasia and cancer therapy are needed. More studies are also necessary to evaluate the risk of bleeding in patients taking DOACs and suffering from gastrointestinal or genitourinary cancers, as well as studies assessing possible drug interactions with immunotherapy and with tyrosine kinase inhibitors [37]. Development of new risk models as well as refinement of already in use models are needed in order to provide a more accurate risk stratification for cancer patients receiving chemotherapy or new anticancer therapies.

## 5. Prophylaxis of Central Venous Catheter Thromboembolism

Upper limbs venous thrombosis related to the insertion of central venous catheter in patients on active cancer therapy has long been debated. There are controversial studies about the benefit of thromboprophylaxis: in past decades, some studies showed a statistically significant reduction of VTE with the use of warfarin or LMWH, with an incidence of events in patients non receiving prophylaxis of even 14%. Most recent studies have resized the problem, limiting the incidence of VTE without prophylaxis to 4–5% and attesting a non-statistically significant difference between patients who received thromboprophylaxis and those who received placebo [38]; this is maybe due to greater expertise in the insertion of venous catheters and new less thrombogenic materials. Moreover, the ETHIC study demonstrated a statistically insignificant reduction in thromboembolic events between patients treated with enoxaparin 40 mg and patients treated with placebo [39].

To be noted that at least two meta-analyses highlighted a higher incidence of VTE with peripherally inserted central catheter (PICC line) than with central venous catheter (CVC) [40].

Based on this evidences, a routine thromboprophylaxis with LMWH in patients with central venous catheter is not currently indicated.

## 6. Thromboprophylaxis in Hospitalized Patients with Acute Medical Condition

Oncological diseases, hospital immobilization, sepsis, advanced age, exacerbations of chronic obstructive pulmonary disease (COPD) are risk factors for the development of VTE in hospitalized patients, as well as personal history of previous VTE. Without thromboprophylaxis, incidence of VTE in hospitalized patients ranges from 10% to 40%, with most of the events occurring after discharge [41].

Three clinical studies assessed the efficacy of primary thromboembolic prophylaxis in hospitalized cancer patients confined to bed with an acute medical complication: the MEDENOX [42] and the PREVENT [43] study evaluated the use of enoxaparin and dalteparin, the ARTEMIS study [44] the use of fondaparinux, with a percentage of cancer patients in these studies of about 10–15%.

In the MEDENOX study, prophylaxis with enoxaparin 40 mg/die reduced the incidence of VTE to 5.5% compared to 15% of patients treated with placebo or enoxaparin 20 mg/die. A subgroup analysis showed that in cancer patients the use of enoxaparin 40 mg/die reduces VTE events by 60%.

The CERTIFY study compared LMWH vs. unfractionated heparin (UFH) in VTE prevention in hospitalized cancer patients with acute medical event, highlighting equal effectiveness and safety [45].

A recent meta-analysis by Carrier et al. showed discordant results, demonstrating that a thromboprophylaxis with LMWH does not statistically reduce the risk of VTE (RR 0.91, 95% CI 0.21–4.0), despite some limitations such as the heterogeneity of the studies and the low sample size [46].

In contrast, another recent meta-analysis showed that in hospitalized cancer patients with other risk factors, the use of LMWH significantly reduced VTE risk (RR 0.32, 95% CI 0.14–0.71) [47].

In conclusion, current guidelines recommend preventive use of LMWH or fondaparinux in hospitalized cancer patients with an acute medical complication. Currently, there is no strong data to support the use of DOACs and the duration of prophylaxis is also an unsolved hot topic.

## 7. Post-Surgical Thromboprophylaxis

In the literature, patients with active cancer undergoing surgery have been reported to have almost twice the risk of experiencing VTE compared to non-cancer patients (37% vs. 20% using fibrinogen uptake test in a study published in 1970) with a quadrupled risk of fatal pulmonary embolism [48,49]; more recently, the risk of VTE following surgery in oncological patients has been resized, thanks to new surgical techniques, new detection methods, and especially to the introduction of pharmacological and mechanical VTE prophylaxis [50]. Various studies compared the safety and efficacy of unfractionated heparin with LMWH in this setting of patients, demonstrating equal efficacy and greater manageability for LMWH [51,52,53,54,55]; particularly a randomized multicenter trial of patients undergoing elective pelvic or abdominal oncological surgery [56] showed that enoxaparin 40 mg/die vs. UFH at low dosage were equivalent in terms of safety and efficacy in reducing the incidence of VTE.

Therefore, in patients undergoing oncological surgery, thromboprophylaxis with LMWH, UFH or fondaparinux, associated with the use of graduated compression stockings, should be taken into account, with LMWH as first choice thanks to the greater manageability (once-daily administration) and the lower risk of heparin-induced thrombocytopenia. There are currently no data on the use of DOACs in this setting of patients.

For what concerns the duration of treatment, ENOXACAN II study evaluated efficacy of enoxaparin 40 mg/die for one week vs. the same dose for four weeks after surgery, in patients undergoing abdominal or pelvic oncological surgery. Results showed that prolonging thromboprophylaxis reduces the risk of VTE from 12% to 4.8% (RR 60%, 95% CI 0.1–0.82) [56,57]. Further studies with different LMWH confirmed these data [58,59,60].

A recent meta-analysis by Bottaro et al. showed that a 4–5 weeks prophylaxis reduces the risk of deep vein thrombosis of 53% compared to one week prophylaxis, with a similar hemorrhagic risk [61].

In conclusion, current guidelines recommend thromboprophylaxis of the duration of at least 7–10 days after both laparotomic and laparoscopic cancer surgery, to be extended up to four weeks especially in case of additional risk factors such as prolonged immobility, advanced age, obesity or previous personal history of VTE [34,62,63].

## 8. VTE Therapy

For what concerns the treatment of VTE in cancer patients, it is important to distinguish two phases of the disease: an acute phase, with the initial treatment and a late phase, with the maintenance treatment.

The initial treatment of the acute phase of VTE in oncological patients (first 5–10 days of therapy) involves the administration of LMWH in single-dose or in double-daily administration based on body weight or UFH in initial bolus of 5000 IU followed by continuous infusion, modulated in order to obtain a PTT ratio of 1.5–2.5 times the basal value. LMWH demonstrated the same efficacy as UFH in the initial treatment of VTE in both non-oncological and oncological patients [64,65,66]. In addition, a recent meta-analysis showed that LMWH is associated with a 3-month reduction in mortality compared to UFH [67,68,69].

Fondaparinux can also be used for the initial treatment of established VTE in patients with cancer. Among the advantages of this drug, its administration contributes to an increased manageability for possibly long-lasting outpatient therapies.

In conclusion, LMWH can be considered the standard of care in the initial treatment of VTE in oncological patients, also thanks to its manageability, while UFH and fondaparinux represent valid second line alternatives.

To be remembered is that LMWH is contraindicated in patients with severe renal failure (i.e., with creatinine clearance <30 mL/min); in those cases, UFH is preferred.

The select-D study verified how rivaroxaban is a good alternative in the treatment of the acute phase of VTE in most oncological patients, while leading to an increased risk of gastrointestinal and genitourinary bleeding which must be balanced and evaluated with each patient based on its advantages (i.e., oral intake, etc.) [65].

For patients who do not have a high risk of gastrointestinal or genitourinary bleeding, rivaroxaban or edoxaban (the last one after at least five days of parenteral anticoagulation) can be used for the initial treatment of established VTE in patients with cancer [30].

A frequent problem in oncological patients is represented by the thrombocytopenia which can be due to chemotherapy, radiation therapy, bone marrow invasion or disseminated intravascular coagulation. The presence of thrombocytopenia is associated with an increased bleeding risk but does not appear to be protective towards thromboembolic events [9].

Therefore, the decision to start heparin in thrombocytopenic patients must take into account several factors including platelet count, recurrence risk, additional hemorrhagic risk factors (liver or renal function alterations, brain metastases, etc.). Based on retrospective analyses and case series, a full-dose treatment with platelet count >50 × 10⁹ L is generally suggested, thus considering the suspension for values <25 × 10⁹ L [70,71]. Among patients with platelet count ranging from 25 × 10⁹ L to 50 × 10⁹ L, the decision to administer heparin should consider other bleeding risk factors. Overall, it is usually suggested to administer anticoagulants without reaching the full expected dose.

For what concerns the treatment of the maintenance phase, numerous studies evaluated the efficacy and safety of vitamin K antagonists (VKAs) and DOACs vs. LMWH. In cancer patients undergoing chemotherapy, the use of VKAs is associated with a greater bleeding risk and a greater risk of thromboembolism recurrence. LMWH remains the first choice in the 3–6 months treatment of VTE in oncological patients, also thanks to its lower half-life that allows rapid dose adjustments (for example in case of bleeding or invasive maneuvers); the VKAs are not easy to handle also due to interactions with chemotherapy that can make it difficult to keep international normalized ratio (INR) in range.

DOACs demonstrated the same efficacy and a better safety profile as VKAs in the prolonged treatment of VTE in some randomized clinical trials; in these studies, however, cancer patients were a small minority. Two studies evaluating the use of DOACs versus LMWH in cancer patients with VTE have recently been published: the Select-D study (rivaroxaban vs. dalteparin) and the HOKUSAI VTE-cancer study (edoxaban vs. dalteparin) [72,73]. In addition, recently a review has been conducted assessing the efficacy and safety of DOACs, LMWH and VKAs in cancer patients affected by VTE [74]. From these studies emerged that DOACs are effective in preventing VTE recurrence but are associated with an increased risk of bleeding compared to LMWH. Therefore, the choice of the anti-coagulant therapy must be modulated for each patient also on the basis of the primary tumor site (for example gastrointestinal or genitourinary) and of patients’ preferences.

Another study has recently been published about the comparison of apixaban vs. dalteparin in cancer patients with acute venous thromboembolism: in the Caravaggio study, a prospective, randomized, non-inferiority clinical trial, patients were randomized to receive oral apixaban or subcutaneous dalteparin for six months. Apixaban was administered at a dosage of 10 mg twice daily for the first week and then five milligrams twice daily, while dalteparin was given at a dosage of 200 IU/kg for the first month and then 150 IU/kg once daily. The primary endpoint of the study was recurrent VTE and the primary safety outcome was major bleeding. Recurrent venous thromboembolism occurred in 5.6% of patients in the apixaban group and in 7.9% of patients in the dalteparin group (HR 0.63, 95% CI 0.37–1.07, *p* < 0.001). Major bleeding occurred in 3.8% of patients in the apixaban group and in 4% of patients in the dalteparin group (HR 0.82, 95% CI 0.40–1.69, *p* = 0.60). In conclusion, apixaban was found to be non-inferior to dalteparin for the treatment of venous thromboembolism in cancer patients without an increased risk of major bleeding [75,76].

Results concerning major bleeding risk are in contrast with other recent studies, where it was found a higher incidence of major bleeding in patients taking DOACs than LMWH. On the other hand, episodes of nonmajor bleeding were numerically higher in the apixaban group, consistently with previous studies (see also Table 3).

The updated ASCO guidelines recommend the use of LMWH, UFH, fondaparinux or rivaroxaban as initial treatments of VTE in cancer patients, while LMWH, edoxaban or rivaroxaban are preferred for the maintenance phase; however, the use of DOACs should be balanced considering the bleeding risk especially in patients with gastrointestinal cancers and in patients with important polypharmacy, due to the risk of drugs interactions [33,34].

In late 2019, an update of clinical practice guidelines from International Initiative on Thrombosis and Cancer working group have been published. The most important novelty is that DOACs are recommended for the maintenance treatment of VTE in cancer patients with creatinine clearance ≥30 mL/min. To keep in mind is the risk of drug interactions and the bleeding risk, which is higher than with LMWH, so caution must be used with patients with gastrointestinal tract malignancies, especially because of data suggesting increased bleeding risk in patients treated with edoxaban and rivaroxaban [30].

There is much debate about the optimal duration of anticoagulant treatment after a first episode of VTE in oncological patients as well as in the general population. Usually it is advised to continue the anticoagulation for the entire duration of cancer treatment unless there are contraindications, with frequent re-evaluations of each patient case in order to ensure that the risk-benefit ratio is still favorable (see also Table 4).

In conclusion, in oncological patients affected by venous thromboembolism, long term therapy (6 months) with LMWH or apixaban/edoxaban/rivaroxaban should be evaluated, and this therapy should be preferred to VKAs.

Regarding patients with recurrent VTE during anticoagulation treatment, a possible approach may be switching from one drug to another (i.e., switch from LMWH to DOACs, from DOACs to LMWH, from AVK to LMWH or DOACs) or increasing dosage of low molecular weight heparin by 20–25% [30].

However, ad hoc prospective studies are needed in the setting of oncological patients on active treatment experiencing VTE, in order to evaluate the use of anticoagulants beyond six months and the interactions in terms of major bleeding, impact on recurrence of VTE and mortality.

## 9. Anticoagulant Therapy and Impact on Disease Prognosis

In the last few years, some retrospective studies assessed the impact of anticoagulant therapy on the prognosis of cancer patients. Two systematic reviews of the studies in the literature provided nonunivocal results [79,80]. On the contrary, a meta-analysis of the studies that investigated the efficacy of UFH and LMWH in patients affected by VTE showed a reduction in mortality in patients treated with LMWH [81].

Three ad hoc prospective studies (MALT, FAMOUS and a study by Altinbas et al. on SCLC patients) support this hypothesis [77,78,82]. The CLOT study also highlighted that the use of LMWH in secondary thrombosis prophylaxis improves the prognosis of patients with initial stage disease compared to VKAs [21]. On the contrary, the IMPACT study did not demonstrate any benefit from the use of LMWH [83].

An overall evaluation of these studies seems to support the hypothesis that the use of LMWH can improve the outcome of patients, in particular those with non-advanced disease.

However, there are numerous critical issues regarding these trials (different doses of LMWH in the various studies, different chemotherapy schemes, nonuniformity in patient selection), and therefore at the present time the predictive role of LMWH in this setting remains to be defined.

## 10. Observations and Future Research Perspectives

Several meta-analyses and reviews have been conducted about VTE prophylaxis and treatment in the last decades. Important limitations of these studies are heterogeneity of cancer types and staging, variability in cancer treatments, different antithrombotic drugs and doses, potential interactions with other patients’ drugs, presence of comorbidities. Due to these limitations, data can result weak and poorly consistent between different meta-analyses.

The use of VTE prophylaxis is currently recommended in cancer patients admitted to hospital for an acute medical condition, but we still do not have sufficient information about the risk of bleeding during thromboprophylaxis. Concerning the thromboprophylaxis in ambulatory cancer patients receiving oncological treatment, refinement of existing VTE risk models or development of new models are needed in order to improve risk stratification of these patients.

The latest guidelines have introduced recommendations about the use of edoxaban and rivaroxaban for treatment of VTE in patients with cancer, but we still need information and experience about real-world use of DOACs in cancer patients, especially for what concerns drug interactions and bleeding risk.

Hence, the management of venous thromboembolism in patients with cancer remains a challenge. Further studies about cancer-associated thromboembolism are ongoing, in order to refine our knowledge concerning the management of VTE therapy and prophylaxis in this delicate setting of patients.

## 11. Conclusions

Cancer patients have a higher risk of developing venous thromboembolism compared to general population, and this is due to several factors such as production of procoagulant factors by tumor cells, administration of chemotherapy and hormonotherapy, hospitalization, systemic inflammatory status and tumor compression stasis.

Several clinical studies about thromboprophylaxis in outpatients receiving systemic treatment for cancer were conducted. Anti-thrombotic prophylaxis seems to be not necessary in all unselected cancer patients on active oncological therapy, whereas prophylaxis with LMWH, apixaban or rivaroxaban should be considered for cancer outpatients who receive chemotherapy and who are at high thromboembolic risk. Current guidelines also recommend preventive use of LMWH or fondaparinux in hospitalized cancer patients with an acute medical complication. Differently, routine thromboprophylaxis with LMWH in patients with venous central catheter is not indicated, but a thromboprophylaxis of at least 7–10 days should be administered after cancer surgery, to be extended up to four weeks especially in presence of additional risk factors.

For what concerns the therapy of manifested VTE in cancer patients, several studies have been conducted; in oncological patients affected by venous thromboembolism, long term therapy (6 months) with LMWH or edoxaban/rivaroxaban/apixaban should be evaluated, and this therapy should be preferred to VKAs. There is much debate about the optimal duration of anticoagulant treatment, and usually it is advised to continue it for the entire duration of cancer treatment unless contraindications.

However, ad hoc prospective studies are needed in the setting of thromboprophylaxis and of therapy of manifested VTE in oncological patients, in order to evaluate safety and efficacy of different anticoagulants, optimal duration of therapy and possible interactions with chemotherapy, immunotherapy and targeted therapy, as well as correlation with patients’ outcome.

## Figures and Tables

**Table 1:** Virchow triad: factors contributing to thrombosis.

Virchow Triad
Blood stasis
Endothelial injury or vessel walls injury
Hypercoagulability

**Table 2:** Khorana score risk factors: predictive model for chemotherapy-associated venous thromboembolism (VTE) [14]. (from Khorana, A.A.; Kuderer, N.M. Development and validation of a predictive model for chemotherapy-associated thrombosis. Blood 2008, 111, 4902–4907).

Factors	Points
**Primary cancer site**	
**Pancreas, stomach**	2
**Lung, renal, bladder, testicular, lymphoma, gynecologic**	1
**Hemoglobin < 10 g/dL or use of red cell growth factors**	1
**Leukocytes > 11.000/µL**	1
**Platelets ≥ 350.000/µL**	1
**BMI ≥ 35 kg/m²**	1
**Interpretation:**	
**High-risk score** **Intermediate-risk score** **Low-risk score**	≥3 points 1–2 points 0 points

**Table 3:** Summary of characteristics and results of the main clinical trials cited in the text on prevention and treatment of VTE in cancer patients.

Trial	Study Design	Setting	Patients’ Disease Characteristics	Anticoagulant Drug	Duration (Months)	Number of Patients	Thromboembolic Events	Major Bleeding
**Carrier et al. (2019) [25]**	Randomized Double blind	Prevention	Khorana score ≥2 cancer patients starting chemotherapy	Apixaban 2.5 mg BID vs. placebo	6	288/275	4.2% apixaban 10.2% placebo	3.5% apixaban 1.8% placebo
**Khorana et al. (2019) [26]**	Randomized Double blind	Prevention	Khorana score ≥2 cancer patients starting chemotherapy	Rivaroxban 10 mg OD vs. placebo	6	420/421	6% rivaroxaban 8.8% placebo	2% rivaroxaban 1% placebo
**Agnelli et al. (2009) [77]**	Randomised Double blind	Prevention	Metastatic or locally advanced solid cancer patients receiving chemotherapy	Nadroparin 3800 IU OD vs. placebo	4	779/387	2% nadroparin group 3.9% placebo group	0.7% nadroparin group 0% placebo group
**Haas et al. (2012) [78]**	Two randomised Double blind	Prevention	Metastatic breast cancer or stage III/IV lung cancer patients	Certoparin 3000 IU OD vs. placebo	6	447/453	TOPIC-1: 4% certoparin 4% placebo TOPIC-2: 4.5% certoparin 8.3% placebo	TOPIC-1: 1.7% certoparin 0% placebo TOPIC-2: 3.7% certoparin 2.2% placebo
**Agnelli et al. (2020) [76]**	Randomized Open label	Treatment	Cancer patients with VTE	Apixaban 10 mg BID for the first 7 days, then 5 mg bid vs. dalteparin 200 IU/kg OD for the first month, then 150 IU/kg OD	6	576/579	5.6% apixaban 7.9% dalteparin	3.8% apixaban 4% dalteparin
**Young et al. (2018) [72]**	Randomized Open label	Treatment	Cancer patients with VTE	Dalteparin 200 IU/kg OD for 1 month, then 150 IU/kg OD vs. rivaroxaban 15 mg BID for 3 weeks then 20 mg OD	6	203/203	11% dalteparin 4% rivaroxaban	4% dalteparin 6% rivaroxaban
**Raskob et al. (2018) [73]**	Randomized Open label	Treatment	Cancer patients with VTE	LMWH for at least 5 days then edoxaban 60 mg OD vs. dalteparin 200 IU/kg OD for 1 month then dalteparin 150 IU/kg OD	6–12	522/524	7.9% edoxaban 11.3% dalteparin	6.9% edoxaban 4% dalteparin

VTE = venous thromboembolism, BID = twice daily, OD = once daily, IU = international unit, VTE = venous thromboembolism.

**Table 4:** Recommendations from international guidelines on anticoagulant treatment of established VTE in cancer patients.

Guidelines	Initial Treatment	Maintenance Treatment	Duration
**ESMO 2011**	Weight-adjusted LMWH or UFH. Monitor anti-Xa activity if creatinine clearance is <25–30 mL/min.	LMWH or VKA.	≥3–6 months; the optimal duration should be individually assessed. In palliative setting, an indefinite treatment should be proposed.
**NCCN 2011**	Weight-adjusted LMWH, UFH or fondaparinux.	LMWH (preferred for the first six months as monotherapy) or VKA.	3–6 months for DVT and 6–12 months for PE. In patients with active cancer or persistent risk factors, indefinite treatment.
**ASCO 2015**	LMWH is recommended for the initial 5–10 days.	LMWH.	six months.
**ACCP 2016**	LMWH is suggested over VKA or DOAC.	LMWH is suggested over VKA or DOAC.	For at least three months, but extended anticoagulation is recommended in patients with active cancer.
**ITAC 2019**	First 10 days: LMWH is recommended; UFH, fondaparinux, DOAC can be also used.	LMWHs is preferred over VKA. DOAC can be considered.	three to six months, then termination or continuation should be based on individual benefit-to-risk ratio.
**ASCO 2019**	LMWH, UFH, fondaparinux or rivaroxaban can be used.	LMWH, edoxaban or rivaroxaban are preferred options.	≥six months. Continuing anticoagulation beyond six months should be considered for selected patients.

ESMO—European Society for Medical Oncology; NCCN—National Comprehensive Cancer Network; ASCO—American Society of Clinical Oncology; ITAC—International Initiative on Thrombosis and Cancer; ACCP—American College of Chest Physicians; LMWH—low molecular weight heparin; UFH—unfractioned heparin; VKA—vitamin K antagonist; DOAC—direct oral anticoagulant.
